# The importance of the PD-1/PD-L1 pathway at the maternal-fetal interface

**DOI:** 10.1186/s12884-019-2218-6

**Published:** 2019-02-19

**Authors:** Matyas Meggyes, Eva Miko, Brigitta Szigeti, Nelli Farkas, Laszlo Szereday

**Affiliations:** 1 Department of Medical Microbiology and Immunology, University of Pecs, Medical School, 7624 Pecs, 12 Szigeti Street, Pecs, Hungary; 2 Janos Szentagothai Research Centre, 7624 Pecs, 20 Ifjusag Street, Pecs, Hungary; 3 Institute of Bioanalysis, University of Pecs, Medical School, 7624 Pecs, 12 Szigeti Street, Pecs, Hungary

**Keywords:** Pregnancy, Maternal-fetal interface, PD-1, PD-L1, Maternal immunotolerance

## Abstract

**Background:**

Our goal with this study was to investigate the contribution of PD-1/PD-L1 immune-checkpoint pathway to maternal immunotolerance mechanisms.

**Methods:**

Thirteen healthy pregnant women and 10 non-pregnant controls were involved in this project. PBMCs and DICs were isolated from peripheral blood and from decidual tissues. After the characterization of different immune cell subsets, we used fluorochrome-conjugated monoclonal antibodies to measure the expression level of PD-1, PD-L1, NKG2D, and CD107a molecules by flow cytometry.

**Results:**

We measured significant alternations in the proportion of decidual immune cell subsets compared to the periphery. Elevated PD-1 expression by decidual CD8+ T, CD4+ T, and NKT-like cells were also detected accompanied by the increased PD-L1 expression by decidual CD4+ T, Treg, NKT-like and CD56 + NK cell subsets compared to peripheral blood. The cytotoxic potential was significantly higher in PD-1- decidual immune cells compared to the periphery, however we measured a significantly lower cytotoxicity in the decidual PD-1+ CD8+ T cells compared with the peripheral subsets. An activation receptor NKG2D expression was decreased by the PD-1+ CD8+ T subsets in the first trimester compared to non-pregnant condition but the expression level of the decidual counterparts was significantly elevated compared to the periphery. The cytotoxic potential of decidual PD1/NKG2D double positive CD8+ T cells was significantly decreased compared to the peripheral subsets.

**Conclusions:**

Based on our results we assume that PD-1/PD-L1 pathway might have a novel role in the maintaining of the local immunological environment. Accompanied by NKG2D activating receptor this checkpoint interaction could regulate decidual CD8 Tc cell subsets and may contribute maternal immunotolerance.

## Background

Pregnancy is a useful model to investigate natural immunotolerance since the semi-allogeneic fetus will neither be attacked nor rejected by the maternal immune system, but rather successfully accepted by the mother. A healthy pregnancy requires that the maternal immune system recognizes the antigens from paternal origin expressed by the fetus thereby developing maternal immunotolerance against the fetus. A failure in the mechanism of recognition consequently the impaired maternal immune tolerance may result in abnormal pregnancies, such as recurrent spontaneous abortion or preeclampsia. For many years, immunotolerance during pregnancy was described as a Th2-type immune response [1]. An altered cytokine production toward Th2-type cytokines during pregnancy is part of these immunological changes and promotes maternal-fetal tolerance (MFI) [2]. Furthermore, the elevated ratio of natural killer (CD56 + NK) and NKT-like cells at the MFI suggests an important role of both the innate and the adaptive immune system [3].

The transmembrane protein Programmed cell death protein 1 (PD-1) is a member of the B7-CD28 family [4]. As a co-inhibitory molecule PD-1 is expressed by a variety of activated immune cells, including T (CD4+, CD8+, NKT-like and regulatory T (Treg)) cells, B cells, monocytes and dendritic cells [5, 6]. After activation, the receptor expression could be rapidly up-regulated within 24 h by naive T cells [7]. The receptor has been originally identified on exhausted T cells, and in most cases the blockade of PD-1 signaling has been shown to revert the dysfunctional state of exhausted CD8+ T cells [8, 9].

Programmed cell death ligand-1 and ligand-2 (PD-L1 and PD-L2) are members of the B7-CD28 family being the two known ligands for PD-1 [6]. The two PD-1 ligands have different expression patterns, while PD-L1 is expressed in many tissue types, including the heart, spleen, and antigen-presenting cells [10], the expression of PD-L2 is very limited predominantly restricted to macrophages and dendritic cells [11, 12]. However Nagamatsu et al. have shown that decidual stromal cells express both PD-1 ligands in term pregnancy [13]. PD-L1 expression by non-hematopoietic cells [14, 15] and in high levels by tumor tissues and different lymphoma subtypes have also been reported [16].

After binding to its ligands, PD-1 can inhibit autoreactive T, B and effector T cells to induce T cell tolerance and regulate local inflammation [17]. PD-1 regulates T cell homeostasis, it also has an important role in peripheral tolerance and in the prevention of autoimmunity [18, 19]. However, the overexpression of PD-1 can cause T cell dysfunction and exhaustion accompanied by impaired IFN-γ secretion [20]. Therefore this pathway has a significant role in the balance between effective immunity and self-tolerance. Similarly to TIM-3 mediated regulation [21], PD-1 signaling in CD8+ T cells is characterized by the diminish of effector functions [22] and this could play a role in the complex mechanism of peripheral immune tolerance [23].

A growing body of evidence proved the significance of PD-1 and their ligands in a different type of cancers [24–26], and emphasize the therapeutic potential of this pathway in the treatment of patients with progressive metastatic non-small cell lung cancer (NSCLC) [27]. The importance of the PD-1/PD-L1 pathway in transplantation models and autoimmunity was also extensively investigated. Many papers reported that disruption of PD-L1 contributes to graft destruction by the host [28, 29], furthermore the deficiency of PD-1 enhances T cell proliferation and graft rejection [29, 30]. The absence of PD-L1 and PD-L2 facilitates the accumulation of activated lymphocytes and the development of T cell-mediated autoimmunity [12].

NKG2D is an activating receptor expressed by NK and NKT-like cells, but it is also present on CD8+ T cells. After binding to its ligands such as UL16 binding protein 1 (ULBP1), ULBP2, ULBP3, major histocompatibility complex class related molecules A and B (MICA, MICB) it has a costimulatory function and contribute to the cytotoxic activity of these effector cells [31]. Although MICA expression restricted to gastric and glandular epithelial cells, the expression pattern of ULBP appears to be extensive in healthy adult tissues since ULBP transcripts were observed in kidney, prostate, uterus, tonsil, lymph node tissues [32] and trophoblast cells [33]. Currently one of the most efficient immunotherapy is to block the PD/PD-L1 checkpoint inhibitor pathway [34, 35]. A recent study reported a connection between a higher PD-L1 and lower NKG2D ligand expression in a radioresistant cell [36]. Therefore it might be interesting to investigate the co-expression of PD-1 and NKG2D receptors by CD8+ T and NKT-like cells since NK cells do not express PD-1 receptor.

Based on the above-mentioned studies from different aspects, PD-1 and its ligands have a critical role to evolve and maintain peripheral immune tolerance [37], nevertheless much less study has been published in the context of the immunoregulation of PD-1 and its ligands during healthy human pregnancy. An interesting study has been reported that in allogeneic pregnancy a quantitative expansion of alloreactive T cells in parallel with reduced Treg function by blocking the surface PD-L1 resulted in decreased fetal survival. It seems that the PD-L1 promote fetal survival by maintaining adequate Treg function. [38]. This paper draws the attention to the importance of PD-1 in normal pregnancy since the expression of PD-L1 by villous syncytiotrophoblasts and cytotrophoblasts could induce the exhaustion of the PD-1 positive lymphocytes [38].

Investigation of the immune-checkpoint molecules at the MFI is crucial in order to clarify the exact immunological relationship between the mother and the fetus. This knowledge accompanied by epigenetic examinations [39] could help us to understand the background of pathological pregnancy conditions such as preeclampsia and intrauterine growth restriction. One of the biggest challenges in reproductive immunology is to find a proper biomarker or biomarker panel for early diagnosis of preeclampsia. We hope that our results supplemented with previous findings [40, 41] will contribute to understanding the background of this pathological condition.

The aim of this present study was to clarify the contribution of PD-1/PD-L1 immune-checkpoint pathway in the induction of maternal tolerance during healthy pregnancy.

## Methods

## Participants

To investigate the PD-1/PD-L1 pathway 13 healthy women who underwent pregnancy termination for non-medical reasons in the first trimester of their pregnancy were recruited. Our control group was formed by 10 healthy non-pregnant women. None of the women had any significant medical history, current or recent illnesses, or were taking medications. All subjects affected by any kind of pregnancy-related complication and/or infection, alcohol abuse, pre-pregnancy disease, AIDS, in vitro fertilization pregnancies, diabetes mellitus, renal diseases, immune-associated disease and deep vein thrombosis were excluded.

### Lymphocyte separation, cryopreservation, thawing

Peripheral blood mononuclear cells (PBMC) were isolated from heparinized venous blood using Ficoll-Paque density gradient (GE Healthcare, UK). Separated cells were then washed twice in RPMI 1640 medium (Lonza, Switzerland), counted, centrifuged and resuspended in human serum containing 10% DMSO (Sigma-Aldrich, USA) for cryoprotection. Subsequently, the cells were aliquoted in cryovials and stored in a -80 °C mechanical freezer. Thawing was carried out on the day of fluorescent cell labeling as quickly as possible in a 37 °C water bath and DMSO was washed out twice in RPMI 1640 medium.

### Isolation of decidual immune cells (DICs)

DIC were obtained from the decidual tissue from clinically normal women after pregnancy termination in the first trimester. Isolation of DICs from decidual tissue was performed as previously described [42]. Briefly, the deciduas were aseptically isolated from fetal tissues and the chorionic villi under a dissecting microscope. Subsequently, the separated tissue bits were cut into pieces and digested in RPMI 1640 supplemented with 0.5% collagenase type IV (Sigma-Aldrich, USA) at 37 °C for 60 min with gentle stirring on a magnetic stirrer. Following the digestion procedure, the cell suspension was passed through a 100 μm cell strainer with a syringe plunger (Becton Dickinson, USA) to prepare a single cell suspension. Next, for tissue debris elimination the samples were washed in RPMI 1640 supplemented with penicillin and streptomycin (P + S) (Lonza, Switzerland) at 1200 rpm for 10 min. The supernatant was aspirated and the pellet was resuspended and passed through a 70 μm cell strainer (Becton Dickinson, USA). After another centrifugation at 1200 rpm for 10 min, the pellet was resuspended and overlaid on a Ficoll-Paque gradient and centrifuged at 2000 rpm for 20 min. DICs were collected, washed, filtered via a 40 μm cell strainer (Becton Dickinson, USA) and resuspended in RPMI 1640 medium containing 10% fetal bovine serum (Gibco, USA), penicillin and streptomycin. Cryoprotection and thawing procedures were performed as previously described.

### Antibodies

For surface and intracellular staining, freshly thawed PBMC and DICs were used. The following monoclonal antibodies were used: fluorescein isothiocyanate (FITC)-conjugated anti-human CD4 (BD Biosciences, USA), FITC-conjugated anti-human CD107a (BD Biosciences, USA), phycoerythrin (PE)-conjugated anti-human PD-1 (Beckmann-Coulter, USA), PE/Cy7-conjugated anti-human NKG2D (BD Biosciences, USA), allophycocyanin (APC)-conjugated anti-human CD56 (BD Biosciences, USA), APC-conjugated anti-human FoxP3 (eBioscience, USA), APC/H7-conjugated anti-human CD8 (BD Biosciences, USA), Brilliant Violet (BV421)-conjugated anti-human PD-L1 (BD Biosciences, USA), BV510-conjugated anti-human CD3 (BD Biosciences, USA).

### Intracellular staining

FoxP3 Staining Buffer Set (eBioscience, USA) was used according to the manufacturer’s instructions to identify the Treg population. Briefly, cells were permeabilized in 1 ml fixation/permeabilization buffer (Concentrate/Diluent 1:4) for 1 h at 4 °C for in the dark. Next, the samples were washed twice in the buffer and stained with the APC-conjugated anti-human FoxP3 monoclonal antibody for 1 h at 4 °C in the dark. The cells were washed twice in the buffer, fixed with 1% PFA and stored at 4 °C in complete darkness until FACS analysis.

### Flow cytometry

Cell-surface expression of specific markers was measured in different immune populations using fluorochrome-conjugated monoclonal antibodies. 10^6^ cell in 100 μl phosphate-buffered saline (PBS)/tube was incubated for 30 min at room temperature with the fluorochrome-labeled monoclonal antibodies according to the manufacturer’s protocol. After a washing step with PBS, the cells were fixed in 300 μl PBS containing 1% paraformaldehyde (PFA) and stored at 4 °C in complete darkness until FACS analysis. Flow cytometer settings were checked using Cytometer Setup and Tracking beads (BD Biosciences, USA) according to the manufacturer’s protocol. To determine compensation values, spectral overlap values are measured for all fluorophores, via single-color controls. Data acquisition and analyses were performed by FACS Canto II flow cytometer (BD Biosciences, USA) equipped with the FACSDIVA V6. software (BD Biosciences, USA).

### CD107a degranulation assay

CD107a surface marker expression was measured by flow cytometry to evaluate the cytotoxic activity of CD8+ T cells. PBMC were incubated in the presence of FITC-conjugated anti-human CD107a monoclonal antibody in RPMI 1640 medium containing 10% fetal bovine serum, P + S, ionomycin (Sigma–Aldrich, USA) and phorbol myristate acetate (Sigma–Aldrich, USA) for 4 h at 37 °C in an atmosphere containing 5% CO_2_. Following the stimulation, cells were washed and resuspended in PBS then incubated with BV510-conjugated anti-human CD3, APC-H7-conjugated anti-human CD8 together with PE-conjugated anti-human PD-1 and PE/Cy7-conjugated anti-human NKG2D antibody for 30 min at room temperature. After the staining, the cells were washed in PBS, fixed with 1% PFA and stored at 4 °C in complete darkness until FACS analysis.

### Statistical analysis

Statistical significance was determined using paired and unpaired sample t-test with statistical software SPSS version 23 package. Normality of our data was tested by Shapiro-Wilk test. A normal distribution was confirmed. Since we didn’t have endometrial tissues from non-pregnant women, statistical comparison was performed using unpaired sample t-test only between PBMC from both groups and never between DIC and PBMC from the non-pregnant group. Data obtained from the same healthy pregnant individuals investigating DIC and PBMC were analysed using paired sample t-test. Differences were considered significant if the *p* value was equal to or less than 0.05.

## Results

### Phenotypic analyses of peripheral and decidual immune cell populations in 1st-trimester healthy pregnant women and peripheral immune cell populations in non-pregnant women

**Fig. 1:**
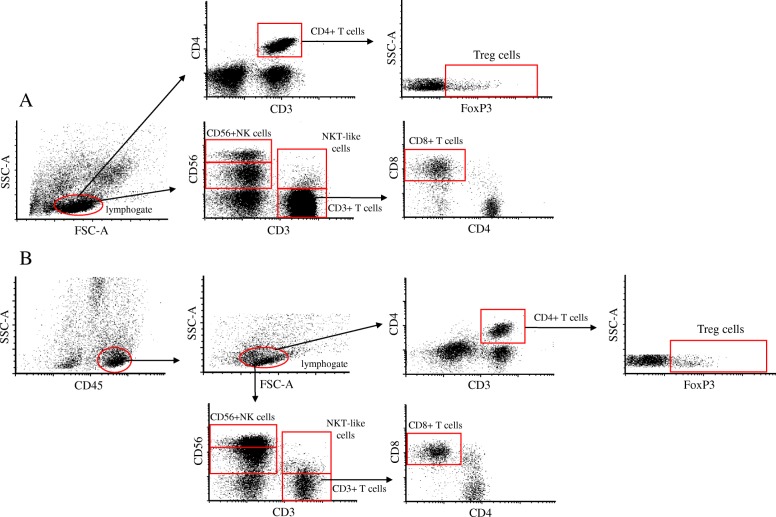
Flow cytometry gating strategy for peripheral and decidual immune cell subpopulations **a**, Lymphocytes from peripheral blood were gated on FSC-A versus SSC-A. Cell surface antibodies were used to identify, CD8+ T, CD4+ T, Treg cells, CD56 + NK, and NKT-like cell subpopulations. **b** Immune cells from decidual tissues were gated using side-scatter area (SSC-A) and CD45 gate. Decidual lymphocytes were selected from CD45+ cells on the basis of forward-scatter area (FSC-A) and SSC-A. Cell surface antibodies were used to identify CD8+ T, CD4+ T, Treg cells, CD56 + NK, and NKT-like cell subpopulations

In our phenotypic examination, different immune cell populations from peripheral blood and from the decidual tissue were compared (Fig. 1). Firstly, we observed a significant elevation in the ratio of the decidual CD8+ T cell subpopulation in parallel with a significant decrease in the ratio of decidual CD4+ T cell subpopulation within CD3+ cell population compared to the peripheral counterparts (Table 1). The percentage of the decidual Treg subpopulation were slightly increased compared to the periphery, but it did not reach a significant level. Similarly to our findings many papers previously reported that the ratio of decidual CD56 + NK cells and CD56^dim^NK and CD56^bright^NK cell subsets were significantly elevated compared to the periphery (Table 1). The percentage of the NKT-like cells did not change significantly between the investigated groups (Table 1).

**Table 1:** Phenotype analysis of different immune cell population in healthy pregnant and in non-pregnant women

	*Non-pregnant PBMC*	*1* ^*st*^ *-trimester PBMC*	*1* ^*st*^ *-trimester DIC*	*p-value*
CD8+ T cells in CD3+ cells	33.21 ± 7.27	34.00 ± 6.90	56.60 ± 15.07*	* < 0.01
CD4+ T cells in CD3+ cells	57.28 ± 10.10	52.51 ± 8.96	31.02 ± 16.70*	* < 0.01
Treg cells	2.19 ± 0.77	1.71 ± 0.79	2.95 ± 3.44	NS
CD56+NK cells	18.44 ± 5.61	13.61 ± 5.17	48.45 ± 22.36*	* < 0.01
CD56^dim^ NK cells	16.43 ± 5.23	11.99 ± 4,28	27.30 ± 14.14*	* < 0.01
CD56^bright^ NK cells	2.04 ± 1.12	1.42 ± 1.03	21.17 ± 12.93*	* < 0.01
NKT-like cells	3.55 ± 3.37	3.74 ± 2.15	4.46 ± 3.67	NS
CD8+ T cells in PD-1+ CD3+ cells	41.59 ± 10.23**	31.48 ± 9.09	62.52 ± 15.19*	* < 0.01 ** < 0.03
CD4+ T cells in PD-1+ CD3+ cells	45.06 ± 13.11	47.39 ± 15.24	28.06 ± 15.30*	* <0.02

Statistical comparisons were made by Student’s t-test between non-regnant PBMC vs. 1^st^-trimester PBMC group and 1^st^-trimester PBMC vs. 1^st^-trimester DIC group. The results were expressed as the mean value ± standard deviation (SD). Differences were considered significant when the value of *p* was equal to or less than 0.05. Non-significant (NS)

*significantly differ from 1^st^ trimester PBMC, **significantly differ from 1^st^ trimester PBMC

The percentage of peripheral immune cell populations did not show any significant difference between women from the 1st-trimester and non-pregnant women.

We further analyzed the percentage of CD8+ T and CD4+ T cells in the PD-1+ CD3+ T cell population. The percentage of CD8+ T cells among the PD-1+ CD3+ T cell population was significantly elevated in decidua of 1st-trimester women and in the periphery of non-pregnant women compared to the periphery of 1st-trimester pregnant women. The percentage of CD4+ T cells among the PD-1+ CD3+ T cell population was significantly reduced in decidua of the 1st-trimester compared to the peripheral counterpart of the 1st-trimester (Table 1).

### PD-1 and PD-L1 expression by peripheral and decidual immune cell populations in 1st-trimester healthy pregnant women and peripheral immune cell populations in non-pregnant women

**Fig. 2:**
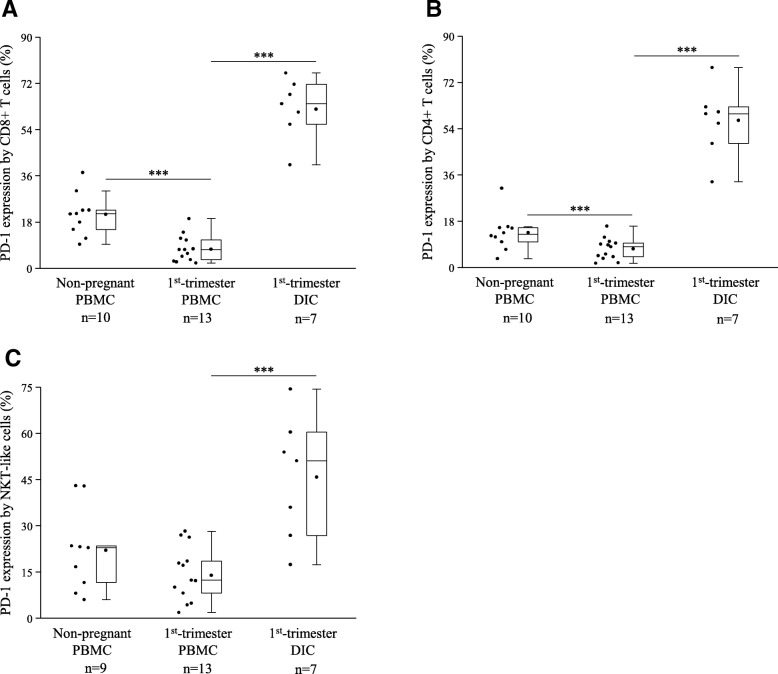
PD-1 expression by different immune cell populations in 1st-trimester healthy pregnant and in non-pregnant women. Box plot of the median, the 25th and, 75th percentiles, range, and individual data values for the expression of the PD-1 receptor by CD8+ T (**a**), CD4+ T (**b**), and NKT-like (**c**) cells in peripheral blood and decidual tissue in healthy pregnant and in non-pregnant women. The middle line within the box represent medians of 10, 13 and 7 determinations, respectively, the middle dot within the box represents the mean, the boxes indicate the interquartile ranges and the whiskers show the most extreme observations. Differences were considered statistically significant for *p*-values ≤ 0.05. ***p < 0,01

Surface expression of PD-1 by CD8+ T, CD4+ T, and NKT-like cells was measured by flow cytometry. The receptor expression was significantly increased in all investigated decidual immune cell subpopulations compared to the peripheral counterparts (Fig. 2). PD-1 expression by peripheral CD8+ T and CD4+ T cells were significantly decreased in the first trimester compared to the non-pregnant condition (Fig. 2a and b).

**Fig. 3:**
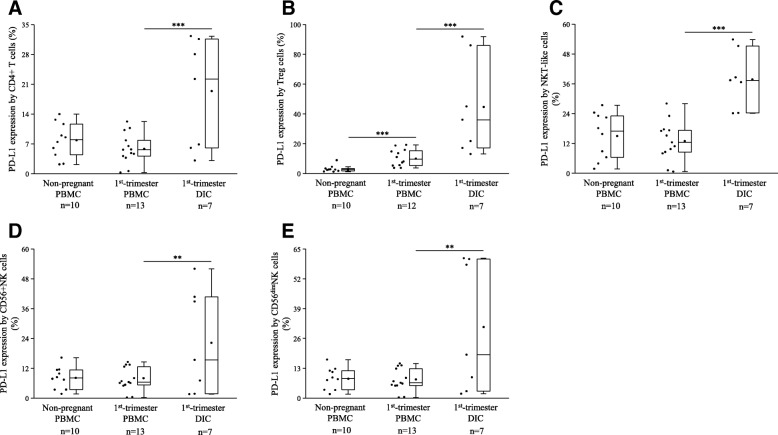
PD-L1 expression by different immune cell populations in 1st-trimester healthy pregnant and in non-pregnant women. Box plot of the median, the 25th and, 75th percentiles, range, and individual data values for the expression of the PD-L1 receptor by, CD4+ T (**a**), Treg (**b**), NKT-like (**c**), CD56 + NK (**d**), and CD56^dim^NK (**e**) cells in peripheral blood and decidual tissue in healthy pregnant and in non-pregnant women. The middle line within the box represent medians of 10, 13 and 7 determinations, respectively, the middle dot within the box represents the mean, the boxes indicate the interquartile ranges and the whiskers show the most extreme observations. Differences were considered statistically significant for *p*-values ≤ 0.05. ***p < 0,01; **p < 0,03

The expression level of PD-L1 shows a unified pattern. The expression of the PD-1 ligand was significantly elevated by all decidual immune cell populations compared to the periphery (Fig. 3.). In the case of the Treg subpopulation, we further detected a significantly increased PD-L1 expression in the 1st-trimester of pregnancy compared to the non-pregnant group (Fig. 3b).

### Cytotoxic activity of PD-1 receptor positive and negative peripheral and decidual immune cell populations in 1st-trimester healthy pregnant women and peripheral immune cell populations in non-pregnant women

**Fig. 4:**
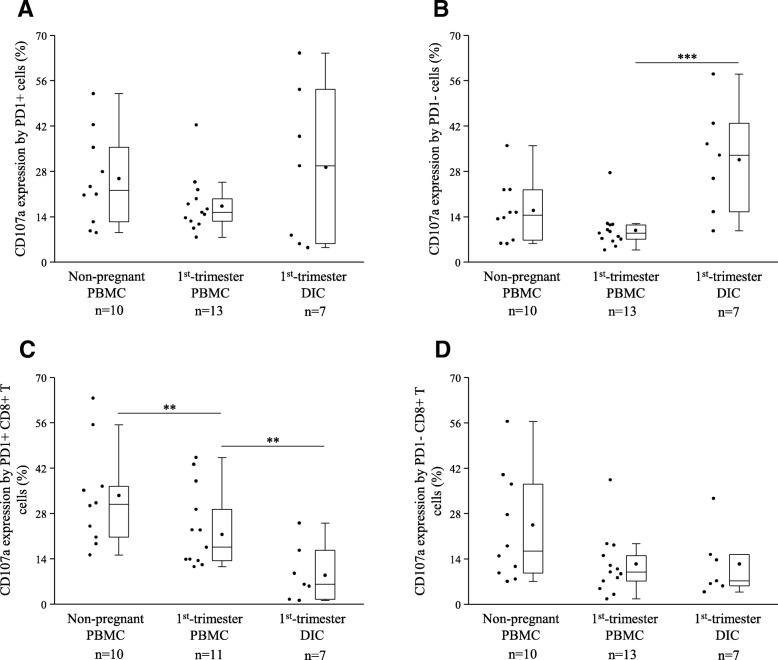
Cytotoxicity of PD-1 positive/negative immune cell populations in 1st-trimester healthy pregnant and in non-pregnant women. Box plot of the median, the 25th and, 75th percentiles, range, and individual data values for the expression of the CD107a molecule by PD-1+/PD-1 negative immune cells (**a** and **b**) and PD-1+/PD-1 negative CD8+ T cells (**c** and **d**) in peripheral blood and decidual tissue in healthy pregnant and in non-pregnant women. The middle line within the box represent medians of 10, 13 and 7 determinations, respectively, the middle dot within the box represents the mean, the boxes indicate the interquartile ranges and the whiskers show the most extreme observations. Differences were considered statistically significant for *p*-values ≤ 0.05. ***p < 0,01; **p < 0,03

To define the cytotoxic potential, CD107a degranulation assay was used. CD107a expression by the PD-1+ cell populations did not show any significant differences between the investigated groups (Fig. 4a). In contrast, decidual PD-1 negative immune cells showed significantly elevated cytotoxicity compared to the peripheral counterpart (Fig. 4b).

Next, we determined the cytotoxic potential of the CD8+ T cells depending on the presence of the PD-1 receptor. Significantly decreased CD107a expression by the decidual PD-1+ CD8+ T cell subset was revealed compared to the peripheral counterparts (Fig. 4c). However, this higher value was significantly lower compared to the non-pregnant control group (Fig. 4c). Notable differences in the cytotoxicity by the PD-1 negative CD8+ T cell populations have not been detected between the examined groups (Fig. 4d).

### Co-expression of NKG2D activating receptor and PD-1 inhibitory immune-checkpoint receptor by CD8+ T cells in peripheral and decidual immune cell populations in the 1st-trimester healthy pregnant women and peripheral immune cell populations in non-pregnant women

**Fig. 5:**
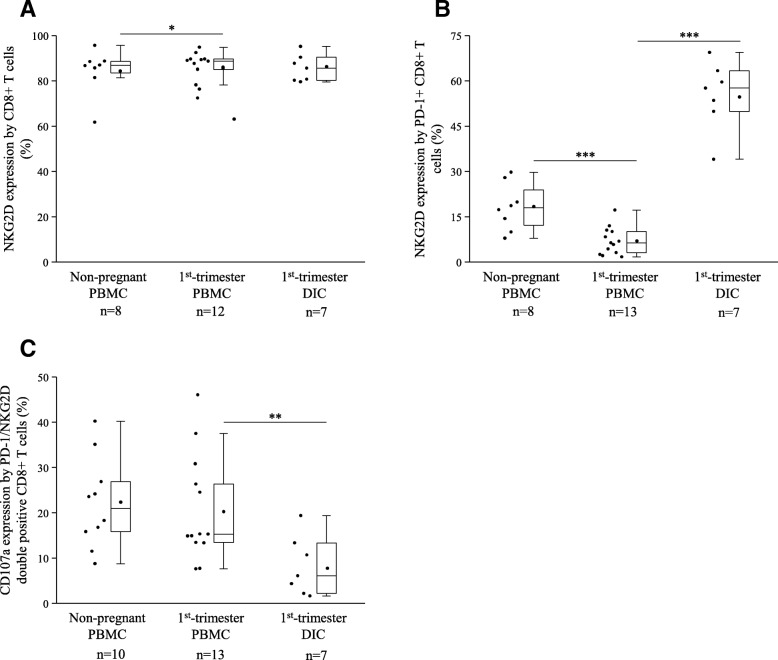
NKG2D expression and cytotoxicity by PD-1+/CD8+ T cells in 1st-trimester healthy pregnant and in non-pregnant women. Box plot of the median, the 25th and, 75th percentiles, range, and individual data values for the expression of the NKG2D receptor by CD8+ T (**a**) cells and PD-1+ CD8+ T cells (**b**) and their cytotoxic potential (**c**) in peripheral blood and decidual tissue in healthy pregnant and in non-pregnant women. The middle line within the box represent medians of 10, 13 and 7 determinations, respectively, the middle dot within the box represents the mean, the boxes indicate the interquartile ranges and the whiskers show the most extreme observations. Differences were considered statistically significant for *p*-values ≤ 0.05. ***p < 0,01; **p < 0,03; *p < 0,05

NKG2D receptor expression by the CD8+ T cells was measured, and we detected a significant elevation in the 1st-trimester healthy pregnant women compared to the non-pregnant group (Fig. 5a). As shown in Fig. 5b, significantly higher NKG2D expression by the decidual PD-1+ CD8+ T cells was observed compared the peripheral PD-1+ CD8+ T cells. Our further analyses detected a significantly decreased NKG2D expression by PD-1+ CD8+ T cells from the periphery in 1st-trimester healthy pregnant women compared to non-pregnant controls (Fig. 5b).

We also measured the cytotoxic potential of the NKG2D/PD-1 double positive CD8+ T cells, and we detected a significantly reduced CD107a expression by the decidual subpopulation compared to the peripheral counterpart (Fig. 5c).

## Discussion

During a healthy pregnancy, the maternal immune system undergoes significant changes in order to tolerate the presence and ensure the proper environment for the fetus. Besides controlling fetal development, the maternal immune system has to react to infections and act against tumor cells. Therefore, abnormalities in this sensitive immunological balance could lead to serious consequences in fetal development including recurrent spontaneous abortion, intrauterine grow restriction or early-onset preeclampsia.

The precise mechanism of this natural immunotolerance against the fetus is still not fully elucidated and exploring the background of these contradictory immune functions could help us to understand the maternal immune homeostasis. Immunoregulation has been realized by the balance of activating and inhibitory immune responses. Checkpoint inhibitors (PD-1, TIM-3, TIGIT, etc.) are one of the key players in the regulation of effector immune responses and cellular interactions preventing from deleterious autoimmune effects, but their significance in maternal-fetal relation is still scarce. The goal of this present study was to evaluate the contribution of the PD-1/PD-L1 immune-checkpoint pathway to maternal immunotolerance during a healthy pregnancy.

To determine the relevance of PD-1 and PD-L1 in pregnancy, firstly we identified several decidual and peripheral immune cell populations, including T- and CD56 + NK cell subsets in the first trimester of pregnancy. There are significant differences in the proportion of the decidual immune cell subsets compared to the periphery. Our phenotypic analyses confirmed that during pregnancy the percentage of decidual CD8+ T-, CD56 + NK-, CD56^dim^NK, and CD56^bright^NK cells were significantly increased compared to the peripheral blood accompanied by a significantly decreased percentage of CD4+ T cells.

There are few publications investigating the presence of PD-1 receptor expressed by immune cells at the human MFI [43, 44]. Taglauer et al. observed an increased presence of PD-1+ CD3+ T cells in the first-trimester decidua compared to the endometrium in non-pregnant controls [44]. Another paper demonstrated an elevated PD-1 expression by the decidual CD8+ T- and CD4+ T cell subpopulation compared to the peripheral counterparts in the first trimester of pregnancy [45]. Consistent with this finding we found that the percentage of decidual CD8+ T cells among in PD-1+ CD3+ T cells was significantly increased compared to the periphery along with the decreased percentage of decidual CD4+ T cells among in PD-1+ CD3 T cells [44]. In line with this result, we further revealed that the percentage of PD-1+ CD8+ T cells significantly decreased in the periphery from the first-trimester of pregnancy compared to the non-pregnant group. One possible purpose for the accumulation of PD-1+ CD8+ T cells at the MFI could be the recruitment of cytotoxic T cells with an anti-microbial effect. At the same time, these cells are under strict control via a PD-1/PD-L1 pathway preventing harmful inflammatory effects.

We also investigated the PD-1 expression by different decidual T cell subsets from the first trimester of pregnancy, and we found and that the receptor expression by decidual CD8+ T, CD4+ T, and NKT-like cells was significantly increased compared to the periphery. Taglauer et al. further reported an elevated PD-1 expression by decidual CD8+ T and CD4+ T compared to the peripheral blood from the third trimester of pregnancy [44]. According to these results, it can be assumed that resident decidual CD8+ T cells express the PD-1 receptor on the surface in a high level during pregnancy ensuring a possible regulation in order to avoid exaggerated inflammatory effects.

The presence of immune-checkpoint ligands on the surface of trophoblast cells was already reported [46]. High PD-L1 expression by syncytiotrophoblast in early and term healthy pregnancy was also published [47] which confirms the importance of this regulatory pathway during pregnancy although there are few data about the presence of PD-L1 ligand on the surface of decidual immune cells. Li et al. presenting information about the expression of PD-L1 by Treg cells but only in mRNA level [43]. Another publication revealed that decidual monocytes express PD-L1 in the first trimester of pregnancy [45]. Our study is the first to investigate the expression level of PD-L1 molecule by different decidual immune cells. According to our analyses, we found a significantly increased PD-L1 expression by CD4+ T, Treg, CD56 + NK, CD56^dim^NK, and NKT-like cells. Besides the presence of PD-L1 in the surface of the syncytiotrophoblast and decidual monocytes, we think that the down-regulation of the accumulated PD-1+ CD8+ T cells in the decidua may occur by the PD-L1 molecule expressed by the Tregs or by CD56 + NK cells locally, which is the most abundant decidual immune subpopulation at the MFI.

After phenotypic analyses, the CD107a degranulation assay was performed to collect further information about the importance of the PD-1/PD-L1 pathway during a healthy pregnancy. Our result showed that CD107a expression by the decidual PD-1 negative immune cells was elevated compared to the periphery, while a significant difference was not observed by the PD-1+ subpopulations. We also investigated the cytotoxic potential of CD8+ T cells depending on the presence of the PD-1 receptor on their surface. We measured a significantly decreased cytotoxic activity by decidual PD-1+ CD8+ T cells compared to the peripheral counterparts. However, we did not find any significant differences in the case of PD-1 negative CD8+ T cells. These data support our theory that PD-1/PD-L1 interaction influences the effector mechanisms of inflammatory immune responses at the MFI already in the first trimester.

An activatory molecule NKG2D was further involved in this study, which is a key receptor on all CD56 + NK cells, including uterine CD56 + NK cells [48] and it is also expressed by CD8+ T cells in a high level. In humans, two families of NKG2D ligands have been discovered, namely MICA, MICB and ULBP1–6 [49]. Liu et al. detected the expression of NKG2D by decidual CD56 + NK cells and ULBP-1 molecule in the surface of syncytiotrophoblast cells [33]. This paper further revealed a relationship between abnormal expression pattern of ULBP-1 by syncytiotrophoblast cells and insufficient invasion ability, which may lead to early-onset preeclampsia [33]. Our study focused on the CD8+ T cell population and their NKG2D expression, which was significantly elevated in the first trimester of pregnancy compared to non-pregnant controls. Recent studies showed that NKG2D ligands could shed from the surface of cancer cells and mediate immunosuppressive function, which causes the lack of immune surveillance against tumor cells [50, 51]. Another paper emphasizes that soluble NKG2D ligands may interfere and could limit the efficacy of immune-checkpoint blockers therapy [52]. It seems that soluble or surface form of NKG2D ligands have a different effect on the receptor-expressing cells. Therefore, we analyzed the co-expression of NKG2D and PD-1 receptors on the surface of CD8+ T cells. The decreased NKG2D expression by PD-1+ CD8+ T cells in the first trimester of pregnancy compared to non-pregnant control accompanied by the elevated NKG2D expression by the decidual PD-1+ CD8+ T cells compared to the periphery may indicate the accumulation of PD-1+ NKG2D^high^ CD8+ T cells at the MFI. Soluble NKG2D ligands from the trophoblast could possibly interact with NKG2D receptors expressed at a high level by local PD-1+ CD8+ T cells. This interaction could play a role in the regulation of these effector cells by reducing their cytotoxic activity via decreased CD107a expression. However, PD-1+ NKG2D^high^ CD8+ T cell interaction via surface NKG2D ligands by the trophoblast could support the invasion of extravillous trophoblast and/or attend in the protection against microbial infections.

There were some limitations to the present study. Firstly, due to technical difficulties, we were not able to separate appropriate amount of cells from decidual tissues to perform all experiments on all samples. Second, functional assays of the investigated cells should be performed in the future to investigate the relationship between PD-1 positive immune cells and PD-L1 molecules.

## Conclusion

The results of this present study demonstrate the complexity of the activating and inhibitory mechanisms at the MFI. Our findings suggest that PD-1/PD-L1 immune-checkpoint pathway could play a novel role in the maintenance of this sensitive immunological balance between the mother and the fetus.

## Abbreviations

DIC: Decidual immune cells
NK: Natural killer cells
PBMC: Peripheral blood mononuclear cells
PD-1: Programmed cell death protein 1
PD-L1: Programmed cell death ligand-1
